# On the Diffusion of Typhoid Fever by Means of Drinking Water

**Published:** 1874-03

**Authors:** Austin Flint


BUFFALO

### Medical and Surgical Journal.

VOL XIII. MARCH, 1874. No. 8.

### Original Communications.

:o:

ART. I.—*On the Diffusion of Typhoid Fever by means of Drinking Water.* By AUSTIN FLINT, M. D.*

Of the late acquisitions in medicine, one of the most interesting and important is the discovery that typhoid fever may be communicated through the medium of water used for drinking or for culinary purposes. It is now less than half a century since the researches of Bretonneau and Louis established the basis of the individuality of this disease. Its non-identity with typhus fever has only within the last quarter of a century been generally admitted, and even now there are some who deny this well-settled doctrine. The contagiousness of typhoid fever has been a mooted question since the date of Louis' researches. It is possible that the doctrine of its non-contagiousness has at the present time some adherents; but the proof of its communicability has been

* To the Editors of the Buffalo Medical and Surgical Journal.

DEAR SIRS.—This paper was read at a meeting of the American Public Health Association, held in the city of New York, in November last. One reason for sending it to you for publication is, that the epidemic which it describes occurred very near Buffalo, and there are certain points relating to the epidemic. which on account of their importance at the time not being appreciated failed to receive attention. These points are, the proximity of the privy of the tavern, and of the privies of the surrounding houses to the well in common use the nature of the soil, the depth of the well and other circumstances bearing on the question as to the pollution with excrementitious matter of the water used for drinking and culinary purposes. Facts with respect to these points may perhaps now be ascertained, and I shall be glad if the publication of the paper in your Journal excites interest in the Physicians living in or near North Boston, sufficiently to lead to enquires which will supply information rendering the history of the epidemic more complete. With much esteem,

Yours very truly, AUSTIN FLINT.

rendered so abundant and conclusive that the number of those who are not convinced of the fact must be exceedingly few. Heretofore, however, some of the ablest of observers and authors have advocated its non-contagiousness. A concise statement of fundamental facts relating to the contagiousness of typhoid fever will prepare for an appreciation of our knowledge touching the agency of water as a medium for the communication of this disease. For the sake of conciseness I shall embody these facts in a few propositions.

1. Typhoid fever is very rarely, if ever, communicated by means of emanations from the bodies of patients affected with the disease.

My own experience in this regard accords with that of numerous observers cited by Murchison, exclusive of an epidemic many years ago, within a circumscribed area, of which I shall presently give an account. I have know of very few instances in which personal intercourse could be suspected as the means of communicating the disease. In these few instances there was room for the supposition either that the disease was not communicated, or that contagion was received otherwise than through the atmosphere. The disease does not spread from cases in the hospitals to fellow-patients, nurses, and medical attendants. This statement is based on large opportunities for observations for more than twenty years. The different members of a family are seldom affected successively in such a way as to show communication of the disease from one to another. Certainly, as a rule, whenever several persons in a family become affected, the most rational, if not an obvious explanation, is that they have been exposed to a cause alike common to all. As a rule, when persons contract the disease in one locality and pass through the disease in another locality they who are brought into contact with, or proximity to, the cases do not contract it. There are exceptions to this rule, but in the exceptional instances there is now a more satisfactory explanation than that which refers the communication by means of an infectious miasm. These are the considerations which establish the first proposition.

2. Isolated cases of typhoid fever are numerous, occurring in situations and under circumstances which preclude the possibility of the disease being due to contagion.

It has been said in contravention of this proposition, that contagion may be operative in these cases, although its sources be not discovered, as, for example, we suppose is the fact when small-pox occurs without our being able to trace it to any exposure. This, however, is not a fair comparison. For the cases are few in which small-pox is not known to be produced by contagion, and hence it is rational to infer the latter, although not known, whereas the number of isolated cases in which typhoid fever occurs without any known exposure in any way is so large that when it is otherwise coincidence or a common causation is to be inferred rather than communicability.

3. Outbreaks of typhoid fever have repeatedly occurred in houses and public institutions in consequence of morbific emanations from sewers, cess-pools, or drains, and from their contents either exposed upon the surface of the ground or permeating the soil. Facts warrant the belief that under certain circumstances the special cause of this disease may be a product of decomposing changes taking place in collections of human excretions. It is claimed that this product always involves a contagium, but in many instances the circumstances are such as to render this not merely improbable, but apparently impossible.

4. Certain outbreaks of typhoid fever are evidently dependent on the importation of cases of the disease, the circumstances being such as to furnish logical proof that the outbreaks are due to the diffusion in some way of a contagion.

In the outbreaks now referred to not only is the disease not produced by local causes alone, but the special cause is a morbific product derived from the bodies of those affected with the disease —a product the efficiency of which depends on its source being the body of a patient having typhoid fever. In other words, the special cause is not an extrinsic poison which patients bring with them, but it is an intrinsic typhoid product—that is, a contagium. An instance, which is perhaps the most remarkable on record, exemplifying the correctness of this proposition, will be presently stated.

These four propositions are submitted as embodying fundamental facts which can be fully established by logical proof. The scope of this paper will not permit me to adduce the proof which might be brought forward for the establishment of each of the propositions. I must, therefore, take it for granted that the facts embodied in the propositions can be fully substantiated. Assuming this, it follows that typhoid fever may or may not be contagious.

Between it and typhus fever there is this difference, namely, typhoid fever is not, like typhus, communicable by means of impalpable emanations from patients affected with the disease. At least, if it be ever communicated in this way, the instances are rare exceptions to the general rule. As a rule, when typhoid fever occurs in isolated cases, there is no proof of its having been caused by contagion—in this respect differing from typhus fever. Typhoid fever may be produced by elements derived from healthy person, which we have no reason to believe is ever a source of typhus fever. But typhoid fever is capable of producing a contagium by means of which the disease is diffused, herein affiliating with typhus fever. Typhoid fever thus may occur sporadically, endemically, and epidemically.

I come now to inquire as to the source of the typhoid fever concontagium. If it be not contained in emanations from the body, it does not, of course, proceed from either the skin or the air passages, and there is certainly no palpable product containing it on the surface of the body. We are, therefore, brought, reasoning by way of exclusion, to seek for it in the alvine dejections. If it be contained in these, by what avenue does it gain entrance into the system? If the dejections containing the contagium are conveyed from dwelliugs by soil-pipes, we can understand that it may pervade the atmosphere of houses, in consequence of defective provisions against the escape of sewer emanations, and if excrementatious matter be deposited on the surface of the ground, the atmosphere within a certain area may be polluted by emanations therefrom, which contain the contagium. But there is logical proof of the diffusion of the disease by contagion under circumstances which render it vastly improbable that the contagium is inhaled; and, therefore, reasoning again by way of exclusion, we are brought to consider the alimentary canal as the avenue through which the contagium enters the system. Thus we are rationally led to the conclusion that drinking water is a medium by which typhoid fever may be communicable.

I have spoken of this conclusion as a late discovery. The supposition or theory that drinking water is a vehicle by which the typhoid contagium may be carried into the system is not of very recent date. It was enunciated by Canstott, in Germany, in 1847, and it has been inculcated since an earlier date by Prof. Von Giett, of Munich. Riecke, also a German, author of a treatise on special pathology and theraputics, published in 1852, reported several instances in which outbreaks were traceable to drinking water polluted with sewage. More recently observations have been contributed by British writers, and especially by Dr. Wm. Budd which seem to furnish demonstrative proof of the communicability of the disease in this way.* Budd, however, and others, have contended for the existence of a contagium in the typhoid dejections received into the system either by means of drinking water or atmospherical emanations, as exclusively the cause of the disease. They claim that the dejections contain a virus not less specific than that of small-pox, and that typhoid fever is never produced otherwise than by the introduction of this virus into the system. Facts render such a doctrine untenable. If the propositions which have been stated are correct, communicability through a contagium in the alvine dejections will account for the connection in only a certain proportion of instances.

For the numerous observations on which the causation of typhoid fever by the agency of a contagium in drinking water, in a certain proportion of instances, rests its claims as a recently discovered truth, I must refer to works treating of this subject. I shall content myself in this paper with an account of an outbreak of the disease which came under my observation thirty years ago, ante-dating any publication on the subject. I have alluded already, in this paper, to the outbreak now referred to. It is, perhaps, the most remarkable on record as embracing a com-

My authority for these statements is Murchison. *Vide* work on Fever, second edition, 1873.

bination of circumstances proving, in the first place, the communicability of typhoid fever and, in the second place, rendering vastly probable, if not certain, the communication by means of a contagium contained in drinking water. The circumstances which relate to the latter point are more significant and forcible because at the time of the outbreak, and until within a late period, the agency of drinking water as a vehicle of contagion was not thought of. The facts were recorded and published by me without any idea that water was the medium of the diffusion of the disease. So completely were the circumstances combined with regard to this mode of communication that had they been deliberately planned with the express intention of rendering the proof as convincing as possible, nothing could have been added. Accident fulfilled all the requirements of careful experimentation with a view to test, first, contagiousness, and, second, the correctness of the theory that the contagion may be contained in drinking water, and in this way gain entrance into the system.

The outbreak occurred in 1843, at a place called North Boston, in Erie County, N. Y., situated twelve miles from Lake Erie. The situation at the time mentioned was in every respect salubrious. There were no paludal grounds in the neighborhood. Neither intermittent fever nor any disease had prevailed for several years. Not only was typhoid fever an unknown disease in that particular situation, but in no part of the county had it been known to have occurred up to that time. The fever then and previously indigenous in this section was a mild remittent, or, as it was generally called, bilious fever. The place called North Boston was a small hamlet consisting of a cluster of houses, embracing nine families, all situated within an area of 100 rods in diameter, but the few houses in which the disease occurred were closely grouped around a tavern, the house furthest removed from the tavern being only ten rods distant. Forty-three persons made up the entire community. On the 21st of September, a young man from Massachusetts traveling westward in a stage-coach, (there were no railroads then in that part of the State,) took lodging at the tavern. He had been ill several days, and kept on his journey until he felt unable to proceed further. He remained at the tavern and died on the

19th of October—twenty-eight days after his arrival. From the testimony of two intelligent physicians who attended this patient, it is certain that his disease was typhoid fever. This fever prevailed to some extent in the town in Massachusetts which the patient left when he started on his journey. Between Oct. 14, five days before the date of his death, (Oct. 19,) and Dec. 7, (twenty-one days,) twenty-eight of the forty-three persons comprising this little community, were attacked with fever, and in ten instances the disease proved fatal.

The first person attacked was a son of the inn-keeper, aged sixteen years. A few days afterward, a daughter, aged fifteen years, was attacked, and afterward, in this family, there were five cases, making the whole number seven, (exclusive of the stranger.) In three of these cases the disease proved fatal. A son of the inn-keeper, who lived at a distance, but at the time of the epidemic came to assist his father's family, and occupied with his wife part of a house about four rods from the tavern, was attacked on the 15th of October. Two cases occurred in a family living about three rods from the town, the patients being children aged seven and nine years. In a family living about three rods from the tavern there were seven cases and two deaths. In a family living about ten rods from the tavern were seven cases and five deaths. In a family living three rods from the tavern were four cases, all recovering. One case occurred in a family living within twenty feet of the tavern. Of these six families, in five the heads of the families, that is, the husbands and wives, were living; the sixth family consisting of a widow and son. The widow was about fifty years of age, and the ages of the male heads of the remaining families were between forty and fifty years. The ages of the wives were not much less. All these escaped, a fact which affords an illustration of the diminished liability to typhoid fever after about forty years of age. Of the families which composed this community, three escaped the disease. Of these three families, one lived about forty rods from the tavern, a stream four rods in width intervening. Another family lived at about the same distance. The third family lived only four rods from the tavern. The latter was the only family which escaped of the seven families grouped immediately around the tavern. This family consisted of a man named Stearns, his wife, four children and a boarder.

The escape of two of these three families may be explained by their distance from the tavern ; but how is the exemption of the third family, living only four rods from the tavern, to be accounted for? In connection with this inquiry an important fact may be mentioned. The family of Stearns had quarreled with the family of the inn-keeper, and were on terms of non-intercourse. After the breaking out of the epidemic the members of this family had no intercourse with any of the families in which cases of fever occured. I ascertained from Stearns that no member of his family saw any case of the disease. A reason for this will presently appear.

The facts of this epidemic, as thus far related, prove the importation of the disease, and its diffusion by contagion ; but, in reference to these points, let me recapitulate the facts : "In a small, isolated community, consisting of nine families, seven of which lived within a few rods of each other, a patient affected with typhoid fever is introduced, and, after lingering twenty-nine days dies with this disease. But up to that event the members of this community were free from disease of any kind ; the situation was in every respect healthy, and typhoid fever was unknown in that place and neighborhood. The patient lingered and died at the tavern, which was a place of daily resort for the members of those seven families with a single exception. One family, consisting of several persons, living but four rods from the tavern, was on terms of hostility with the inn-keeper, which precluded all intercourse. The arrival of a sick stranger with a severe disease was an event of interest to the inhabitants. He was visited more or less daily by the different members of the families living close at hand with the exception of one family, and the members of the inn-keeper's family were, of course, brought into close contact with the disease. Twenty-three days after the arrival of the stranger, two members of the family of the inn-keeper were attacked with the disease, and subsequently five others in this family. In all the other families living within a few rods of the tavern, excepting a single family cases occured within about a month, and during this period more than half the population of this little community had been affected. The disease then ceased further progress, no cases afterward occurring. The family in which no case occurred was the only family of the seven families grouped immediately around the town which escaped. The relations of this family to that of the inn-keeper precluded all social intercourse, and, shortly after the disease began to spread, its production, (as will presently be seen,) being imputed to the agency of this family, intercourse of the latter with all the families affected with the disease was at once suspended." This recapitulation is quoted from my report of the epidemic published in 1852 in a work entitled *Clinical Reports on Continued Fever.* This work has for years been out of print. To the extracts just quoted were added the following remarks: "Now in view of this reviewal of the facts if it be claimed that the disease was not transported to the place, and diffused by contagion, it is necessary to admit a series of coincidences almost incredible. The circumstances embrace all the important conditions for a fair experiment in order to test the contagiousness of a disease. In deed, if every circumstance connected with the outbreak of the disease at North Boston had been deliberately selected and arranged for a scientific object they could hardly have been rendered more complete."

An important part of the history of this epidemic remains to be stated. At the time of writing the report from which the foregoing extracts are taken, and for many years afterward, indeed, up to a recent date, I had no idea of the diffusion of typhoid fever through the agency of drinking water. At the time of the epidemic nothing had been published on the topic, and at the time of writing this report and long afterward, I was not aware that any one had entertained this view of the causation; there was nothing relating to it in the medical literature of this country. At that time the non-contagiousness of typhoid fever was enunciated in standard works, among the points showing the non-identity of this fever with typhus. I supposed that the disease at North Boston was communicated by means of a contagium contained in the emanations from the body. I shall now cite from my report certain facts which render vastly probable if not certain, the belief that the disease in this epidemic was communicated through the agency of drinking water. I quote further from my report as follows: "The occurrence of a severe form of disease, affecting in a brief period more than one-half of the small, isolated community, and proving fatal in so large a proportion of cases, as might be expected occasioned not a little excitement at the place. This was naturally occasioned by the fact that the disease was one presenting, for that locality, remarkable features, giving rise to discussion and discrepancy of opinion among the medical practitioners of the neighborhood. The popular explanation of the origin of the disease added greatly to the interest of the excitement. The story was started that the disease was caused by some poison introduced into the families affected through the agency of Stearns whose relations to the inn-keeper were hostile, and whose family alone, of those living close to the tavern, was not affected. The family of the inn-keeper and the other families in the immediate vicinity were in the habit of obtaining the water used for drinking and other domestic purposes from a well near the tavern. It was charged upon Stearns that he had poisoned this well, and that this was the source of the disease. This story was believed by nearly all the inhabitants, so that several pumps were placed in the well, and an effort was made, but without avail, to exhaust all the water which it contained. The family of Stearns alone, of all the families in the immediate vicinity, did not draw water from the tavern well. This family, up to a short time before the outbreak, had obtained water from this well, but owing to the animosity of the inn-keeper, this privilege had been denied to Stearns, so that he was obliged to dig out and deepen an old well of his own. Two other families which escaped, did not get water from the inn-keeper's well owing to their distance from the tavern. By all the other families the water from this well was used daily. These facts coupled with the relations between Stearns and the inn-keeper, and the singular character of the disease, were considered to furnish circumstantial proof of guilt sufficiently conclusive. The charge of poisoning was so openly made that a prosecution for slander was commenced by Stearns, which was finally settled on the payment, by the party prosecuted, of a hundred dollars. Some water which I obtained from the well was examined by chemical reagents and found to be remarkably pure as regards saline constituents. It was not, however, examined for organic matters.

Now, taking into view the statement contained in one of our preliminary propositions, namely, that typhoid fever is rarely, if ever, communicated by means of emanations of the body, together with the observations which within late years point to drinking water as the medium of communicability, that the latter was the mode of diffusion in the North Boston epidemic hardly admits of a doubt; and the circumstances were as remarkably combined with reference to this conclusion as with reference to the proof of contagion. It can hardly be doubted that the exemption from the disease of the family of Stearns was due to the animosity of the inn-keeper, which led the latter to prohibit the use of his well, and compelled Stearns to dig a well of his own. The two families, living forty rods from the tavern, escaped, because, owing to the distance, they did not obtain water from the inn-keeper's well. It may be asked, is it certain that the special cause was a contagium; was not the disease produced simply by excremental decomposition, since facts show that it may be so produced? In other words, may not the disease have been produced by changes occurring in connection with the dejections, without the presence of a contagium? The answer to these questions is this: If due to excremental decomposition without a contagium, why did the disease break out so rapidly shortly after the arrival of the sick stranger? It is far more rational to infer the existence of a contagium than to consider the connection of the epidemic with the arrival of the stranger as a mere coincidence. At the time of the epidemic no suspicion of the presence of the special cause in the drinking-water being entertained, pains were not taken to note the situations of the privies, the nature of the soil, &c. In order to obtain some information on these points I wrote recently to Dr. P. Barber, at the date of the epidemic, and until lately a practitioner in that neighborhood. Dr. Barber writes that, according to his recollection, the privy attached to the inn was situated three or four rods from the well, and he recollects that the contents were allowed to accumulate. The well was by the road-side, supplying with water the inn and the stables, as well as the immediate neighbors.

Another question may arise, namely: Was the evidence conclusive that the disease was typhoid fever? During the progress of the epidemic, when the excitement was at its height, I visited the place on behalf of the county authorities, in order to investigate the nature and origin of the disease. I made an autopsy, noted the history and symptoms in all the cases then in progress, and afterward obtained full records of ten cases. The autopsy disclosed the characteristic intestinal leisons of typhoid fever, and the histories contained the diagnostic features of this disease. The details are given in my report already referred to. The proof that the disease was typhoid fever was as complete as possible.

The discovery of the communicability of typhoid fever by means of a contagium derived from the alimentary canal, while it furnishes a striking point of distinction from typhus fever, yet shows an interesting point of analogy to the latter disease. In typhus the contagium is doubtless contained in the emanations from the body, either in the breath or in the exhalations from the skin or perhaps both, and typhus may be caused, irrespective of contagion, by a morbid matter produced in concentrated emanations from healthy bodies. In typhoid fever, the contagium is in the dejections, and this fever may be and is generally caused by a morbific matter produced in decomposing excrescent from healthy bodies.

As regards prevention the diffusion of typhus contagion is to be avoided by the isolation of cases in respect of those who are susceptible, conjoined with the freest possible ventilation. The spontaneous occurrence of this disease is to be avoided by guarding against overcrowding dwellings or apartments, together with complete ventilation. The diffusion of typhoid fever by contagium is to be avoided by the disinfection of the dejecta from typhoid patients, and by ample protection against the pollution therewith of water or air. The spontaneous occurrence of this disease is to be avoided by complete protection against the pollution of water or air by the dejecta from healthy persons. This involves safeguards, especially in cities, relating to sewers, drains, cesspools, soil-pipes and the waste pipes connected with the latter, as well as to the final disposition of the excrementitious material. These safeguards, in the City of New York, are largely disregarded, and therein is a source of not only typhoid fever, but probably other diseases, the causitive connection of which with this source is not as yet so well established.

Within the past few months the interest and importance belonging to the subject of this paper have been curiously exemplified by the diffusion of typhoid fever through the agency of milk. Several outbreaks in England have been imputed to infected milk; but in the recent instance referred to the proof of this having been the source seems sufficiently conclusive. This outbreak was in one of the healthiest parishes in the West End of London. About 500 cases of typhoid fever were distributed in 104 families in this parish. Of these 104 families, ninety-six were known to have used milk from the same dairy; the facts with regard to the milk supply in the remaining eight families not having been ascertained. It was ascertained that in one of the farms belonging to this dairy there had been cases of typhoid fever, and the sanitary conditions were exceedingly bad. Other details, which I do not introduce, corroborated the conclusion that the diffusion of the disease was due to the milk supply, and no other source was discoverable.*

The infection or the contagium in milk is, of course, derived from the water used in washing the milk-cans, and, perhaps, in the dilution of the milk. The diffusion of the disease in this way therefore, is through the medium of drinking-water.

The discovery of the causation of typhoid fever through this medium naturally has led to the inquiry whether other diseases may not be trace to drinking water which either contains viruses of contagion or is polluted by divers kinds of morbific matter. The facts to which it has been the object of this paper to call attention have opened up a new field for investigation in etiology,

* Vide American Journal of Medical Sciences, number for October, 1873; page 535.

and further researches in this direction may shed much light on the causation of numerous diseases. Already, in the opinion of many, there is ground for assuming that epidemic cholera is diffused by means of a contagium, derived from the alimentary canal, with which drinking water is liable to become infected. This opinion is based on analogical reasoning rather than on logical proof. That water polluted by any kind of morbific matter may prove an exciting or an auxiliary cause of an attack of cholera, during the epidemic prevalence of the disease, is highly probable; but that the disease in this or any other way is communicable, seem to me to be a question concerning which the most to be conceded is that it admits of discussion. To enter upon such a discussion would not be a small undertaking, and I have already occupied as much time as I have a right to appropriate.

